# Effectiveness of incentives and follow-up on increasing survey response rates and participation in field studies

**DOI:** 10.1186/s12874-019-0868-8

**Published:** 2019-12-05

**Authors:** Michael G. Smith, Maryam Witte, Sarah Rocha, Mathias Basner

**Affiliations:** Unit for Experimental Psychiatry, Division of Sleep and Chronobiology, University of Pennsylvania Perelman School of Medicine, 1017 Blockley Hall, 423 Guardian Drive, Philadelphia, PA 19104-6021 USA

**Keywords:** Field study recruitment, Postal questionnaires, Response rate, Cost effectiveness, Public health research, Sleep disturbance, Aircraft noise

## Abstract

**Background:**

Questionnaires are valuable data collection instruments in public health research, and can serve to pre-screen respondents for suitability in future studies. Survey non-response leads to reduced effective sample sizes and can decrease representativeness of the study population, so high response rates are needed to minimize the risk of bias. Here we present results on the success of different postal questionnaire strategies at effecting response, and the effectiveness of these strategies at recruiting participants for a field study on the effects of aircraft noise on sleep.

**Methods:**

In total, we mailed 17 rounds of 240 questionnaires (total *n* = 4080) to randomly selected households around Atlanta International Airport. Different mailing rounds were varied in the length of the questionnaire (11, 26 or 55 questions), survey incentive (gift card or $2 cash), number of follow-up waves (0, 2 or 3), incentive for participating in a 5-night in-home sleep study ($100, $150 or $200), and address personalization.

**Results:**

We received completed questionnaires from 407 respondents (response rate 11.4%). Personalizing the address, enclosing a $2 cash incentive with the initial questionnaire mailing and repeated follow-up mailings were effective at increasing response rate. Despite the increased expense of these approaches in terms of each household mailed, the higher response rates meant that they were more cost-effective overall for obtaining an equivalent number of responses. Interest in participating in the field study decreased with age, but was unaffected by the mailing strategies or cash incentives for field study participation. The likelihood that a respondent would participate in the field study was unaffected by survey incentive, survey length, number of follow-up waves, field study incentive, age or sex.

**Conclusions:**

Pre-issued cash incentives and sending follow-up waves could maximize the representativeness and numbers of people from which to recruit, and may be an effective strategy for improving recruitment into field studies.

## Background

Postal questionnaires are a rather inexpensive and unobtrusive method of data sampling among large study populations, and so are widely used in epidemiological research. Despite their usefulness, a drawback of surveys is the potential for introducing error during the sampling process. There are four main types of survey error: coverage error, when the sample population is not representative of the characteristics the surveyor wishes to estimate; sampling error, when the characteristics of the sampled individuals from a sample population are not representative of the sample population of interest as a whole; nonresponse error, reflecting differences between sampled individuals who do and do not respond to the survey; and measurement error, when survey responses are not accurate reflections of the true value [1]. Utilizing survey questions with high construct validity can reduce measurement error. Coverage and sampling error can be mitigated with appropriate survey design, such as probability sampling. A key aspect of probability sampling is that each individual in the sample frame has an identical probability of being sampled, with the aim of obtaining a sample that represents the whole, unobserved sampled population [2]. Individuals who are absent from the probability sample are termed non-respondents, with the primary reasons for non-response being failure to contact individuals or contacted individuals refusing to respond [3, 4].

Regarding nonresponse error, researchers have commonly used survey response rates as a measure of the quality and representativeness of the data obtained [5, 6]. However, nonresponse bias can occur in surveys both with high and low response rates [7], and the American Association for Public Opinion Research recognize that response rates are not necessarily an indication of data accuracy [8]. This has led some to argue that the representativeness of responses is more important than the response rate per se [9]. Nevertheless, higher response rates can reduce the likelihood of nonresponse error [1], and response rates remain a crucial step towards understanding the presence of survey error [10].

One of the challenges faced by public health research is the current trend for decreasing response rates, often precipitously, to all survey modes [11]. In turn, this leads to reduced effective sample sizes and increased risk of nonresponse bias [1], which could limit the viability of conclusions drawn from the data [12]. Researchers have adopted a number of methods to improve response rates, which include monetary and non-monetary incentives, changes in the length and appearance of questionnaires, different methods of returning completed questionnaires, pre-notification and different approaches to follow-up contact [13]. Response rates to postal surveys can be improved with reduced survey length, the use of incentives and follow-up contact with non-respondents, but these findings are not consistent across different studies [13, 14]. There is also a risk that incentives may introduce bias, by being more appealing to those with lower socioeconomic status [15]. Survey follow-up and incentivization also increases methodological expense, although this may be offset by the reduced need for further sampling from a study population to obtain an equivalent sample size.

One area of public health research that has often utilized survey data is investigations of sleep behaviors among the general population [16]. Sleep is a biological necessity [17], and sufficient quantity and quality of sleep is a vital component of good physical and mental health [18]. Noise can disrupt sleep, with the World Health Organization estimating in 2011 that sleep disturbance by traffic noise accounts for the annual loss of 903,000 healthy life years in Europe alone [19]. Although epidemiological studies on the effects of noise on sleep often use questionnaires to measure sleep disturbance [20], the unconscious nature of sleep makes self-assessment difficult. Furthermore, noise can induce biological responses without cognizance but that may be relevant from a health and wellbeing perspective. To give two such examples, awakenings can be as short as 15 s [21] but are recalled only if they persist for minutes [22], and reported associations between nocturnal traffic noise and increased incidence of cardiovascular disease may be attributable to noise-induced elevations of heart rate and blood pressure during sleep [23]. In addition to questionnaire data on the effects of noise on sleep, physiologic data are therefore needed. As part of an investigation into the potential impact of aircraft noise on physiologic measures of sleep disruption, we conducted an in-home pilot study where we measured sleep and indoor aircraft noise among individuals living close to Hartsfield-Jackson Atlanta International Airport (ATL). Study participants were recruited using postal questionnaires to pre-screen their interest and eligibility for the field study [24]. The objectives of the pilot were to establish the feasibility of unattended acquisition of acoustic and physiologic field data, provide data for sample size calculations, and to determine the postal survey methodology that would most effectively maximize the questionnaire response rate and field study participation rate. This final objective, maximizing response to postal questionnaires, forms the basis of the current paper.

## Method

### Target population

The investigation presented in this paper was a pilot study prior to a larger national study, and was conducted around ATL. Since aircraft noise and its effects on sleep were of interest, we calculated nighttime (23:00–07:00) aircraft noise levels (*L*_night_) around the airport using data from 2014 to 2015 provided by the Federal Aviation Administration (FAA). We modeled nighttime events and calculated the noise levels individually for each aircraft using the FAA’s Integrated Noise Model for 84 nights, validating the result against a 1-year *L*_night_ average from 2012 provided by the FAA. We stratified areas into five noise exposure categories: < 40 dB, 40–45 dB, 45–50 dB, 50–55 dB and > 55 dB. The ATL runways are oriented West-East, so we further subdivided areas into West or East, yielding 10 noise exposure categories.

### Survey protocol

Between September 2016 and July 2017, we sent paper surveys along with a letter of introduction to 4080 randomly selected households around ATL. The introduction letter briefly described the purpose of the survey, informed the recipient that participation was voluntary, assured the confidentiality of their responses, and provided contact information for the research group responsible for conducting the surveys. Also provided was the survey eligibility criteria: 21 or more years of age and only one respondent per household, preferably the adult whose birthday was most recent. Respondents returned surveys by mail using an included pre-paid addressed envelope, or completed them online by following a URL or scanning a QR code.

The primary aim of the survey was to recruit participants for a field study that would measure physiological response to aircraft noise during sleep over five consecutive nights. The surveys indicated the financial compensation that would be awarded for participating in the field study, one of $100, $150 or $200, and included items on whether respondents would be interested in taking part in such a study.

Complete versions of the surveys are provided in the Additional file 1. We developed the surveys especially for this study, based on existing questions designed to measure sleep, noise annoyance, noise sensitivity and sociodemographic data [25–28], with additional questions to assess eligibility for participation in the field study. Surveys differed in length and were characterized as short (11 questions), medium (26 questions) or long (57 questions). The short survey included items on sleep quality and noise-induced sleep disturbance, health, noise sensitivity, ethnicity, sex and age. The medium-length survey further included items on sleep medication, sleep disorders, sleep-promoting coping strategies, hearing acuity, diagnosed hypertension and/or arrhythmia, shift work, residence duration, household children, height and weight. The long survey further included items on habitual sleep and wake times, frequency of sleep difficulties, expanded noise sensitivity, noise annoyance, diagnosis and treatment for an expanded number of medical conditions, marital status, income, education level, employment status and residence sound proofing treatment. The medium and long versions were sufficiently comprehensive to determine whether a respondent met the field study inclusion criteria, but the short survey required us to contact the respondents via telephone for additional information.

Surveys were sent in batches of 240 in seventeen mailing rounds (*n* = 4080). An equal number of surveys were sent to each noise exposure category within each round (24 surveys to each of the 10 noise exposure categories). Mailing rounds differed in the incentive for completing the survey, the length of the survey, the number of follow-up (reminder) waves issued after the initial mailing, and the monetary incentive for participating in the field study if eligible (Table 1). The incentive for completing the survey was either $2 cash included in the initial survey mailing wave, or an Amazon gift card of $2, $5 or $10 value provided upon completion of the survey.

**Table 1:** Overview of each survey round

Round	Incentive for completing the survey	Survey length	Number of follow-up waves	Incentive for participating in field study	Addressee	% deliverable
1	Gift card	Long	0	$100	“Current Resident”	91.3
2	Gift card	Long	0	$100	“Current Resident”	92.9
3	Gift card	Long	0	$100	Personalized	91.7
4	Gift card	Long	0	$100	Personalized	88.8
5	Gift card	Long	0^a^	$100	Personalized	91.3
6	$2 cash	Long	3	$150	Personalized	88.3
7	$2 cash	Long	3	$150	Personalized	89.6
8	$2 cash	Medium	3	$150	Personalized	87.5
9	$2 cash	Short	3	$150	Personalized	86.3
10	$2 cash	Long	3	$200	Personalized	84.6
11	$2 cash	Long	0	$200	Personalized	91.3
12	$2 cash	Long	3	$200	Personalized	85.0
13	$2 cash	Long	3	$200	Personalized	86.3
14	$2 cash	Long	2	$200	Personalized	85.4
15	$2 cash	Long	2	$200	Personalized	84.2
16	$2 cash	Long	2	$200	Personalized	83.8
17	$2 cash	Long	2	$200	Personalized	82.1

^a^Included pre-survey notification postcard sent before the initial survey mailing

Prior to the initial survey wave, a pre-survey notification postcard was sent out only in round 5. Following the initial survey wave within each round, there were 0, 2 or 3 follow-up waves sent if a completed survey had not yet been received from a specific household. The first follow-up, sent 7 days after the initial survey, consisted of a postcard encouraging the recipient to return and complete the original survey if they had not yet already done so. The second follow-up, sent 21 days after the initial survey, consisted of a reminder letter, a new paper copy of the survey and a new pre-paid envelope for returning the survey. The third follow up, sent 42 days after the initial survey consisted of a reminder letter, a further new paper copy of the survey and a further new pre-paid envelope for returning the survey.

Mailing rounds 1–2 were addressed to “Current Resident” and rounds 3–17 were personalized and addressed to a named individual or current resident, for example “A. N. Other or Current Resident”. Rounds 1–2 were mailed in envelopes measuring 24 × 10.5 cm, and rounds 3–17 were sent in 23 × 15.5 cm envelopes. In addition to a University of Pennsylvania logo on the envelope of all mailing rounds, rounds 1–2 indicated that “Perelman School of Medicine, University of Pennsylvania, Department of Psychiatry, Division of Sleep and Chronobiology” sent the mail, and rounds 3–17 indicated only “University of Pennsylvania” as the sender.

The United States Postal Service could not always deliver the surveys to the listed address. We classed a survey as “non-deliverable” if at least one survey, from any wave within a round, was returned to sender. Such reasons for returning to sender included vacant address, unable to be forwarded, incorrect address or reasons unknown. The percentage of surveys that were deliverable within each mailing round are given in Table 1. If a completed survey was received for a recipient that had been classed as non-deliverable (*n* = 9), we reclassified the survey as deliverable. A number of surveys were returned to the sender because the recipient was deceased (*n* = 1), refused delivery of the survey (*n* = 23) or returned a blank survey indicating they were not interested (*n* = 5): these instances were classed as deliverable but as non-response.

### Analysis

We performed statistical analysis in IBM SPSS Statistics (version 25). We excluded surveys that were non-deliverable from all analyses with the exception of analysis of survey delivery rates. Binomial logistical regression models were constructed with completed survey (yes/no), interest in taking part in the field study (yes/no), or participation in the field study (yes/no) as the dependent variables. A number of regression models were constructed, including a combination of survey incentive (gift card/$2 cash), survey length (short/medium/long), number of follow-up waves (0/2/3), field study incentive (150/200), noise exposure category (< 40/40–45/45–50/50–55/> 55 dB) and orientation to the runway (West/East) as nominal predictor variables. Furthermore, sex (woman/man) and age category (18–29/30–39/40–49/50–59/60–69/70+) data from completed surveys were used as predictor variables in a regression model for both interest and participation in the field study. For each model, we performed an overall omnibus test (χ^2^ tests) relative to the intercept-only model, and χ^2^ tests within each model to examine whether there were significant fixed effects for any of the independent variables. Respondents with missing data were excluded from analyses involving the missing variables. Age data were missing for 43 respondents (10.6%), sex data were missing for 21 respondents (5.2%), and interest in the field study data were missing for 5 respondents (1.2%).

The level of statistical significance was set at α = 0.05. Results are reported as odds ratios (OR) and 95% confidence intervals (CI).

We calculated the cost effectiveness of the different survey strategies based on the cost of envelopes (both for mailing the surveys to the study population and the enclosed pre-paid envelopes for returning the completed surveys), paper, color printing, survey incentive and postage. Color printing cost $0.075 per page, with 3 pages for the short survey and 4 pages for the medium and long surveys. Mailing envelopes cost $0.086 each, which also required printing in color. Pre-printed return envelopes cost $0.093 each. We used the current cost of first class postage ($0.50) rather than the cost when we mailed the surveys.

## Results

### Delivery rates

Across all 17 rounds, 3576 out of 4080 surveys (87.6%) were deliverable. A breakdown of the delivery rate, by survey round, is given in Table 1. When the survey was addressed only to “Current Resident”, the mean deliverable rate was 92.1% (95% CI: 89.3–94.2%). When the survey address was personalized, the mean deliverable rate was 87.1% (95% CI: 85.9–88.1%). Regression analysis showed that there were lower odds (OR = 0.578, 95% CI: 0.409–0.817) of delivery to personalized individuals than “Current Resident” only (χ^2^(1,*n* = 4080) = 9.668, *p* = 0.002).

### Survey completion

Out of 3576 delivered surveys, 407 were completed, a response rate of 11.4%. The majority (*n* = 309, 75.9%) were returned by mail, with a minority (*n* = 98, 24.1%) completed online.

Among deliverable surveys within rounds 1–5, there was a 4.3% response rate when addressing the survey to a named individual in larger envelopes that indicated only “University of Pennsylvania” as the sender. The response rate was 1.4% when addressing the survey to only “Current resident” in smaller envelopes that indicated “Perelman School of Medicine” and “Department of Psychiatry, Division of Sleep and Chronobiology” as the sender. The higher response rate among personalized, larger envelope, “University of Pennsylvania” sender surveys was statistically significant (Wald χ^2^(1, *n* = 1094) = 6.772, *p* = 0.009, OR = 3.261, 95% CI: 1.339–7.942).

We performed a regression analysis including the only round with pre-notification (round 5) and the two rounds that were otherwise identical except for pre-notification (rounds 3 and 4). There were higher odds for survey response when issuing a pre-notification postcard (OR = 1.759, 95% CI: 0.821–3.765), but the effect was not statistically significant (Wald χ^2^(1, *n* = 652) = 2.113, *p* = 0.146).

Results of the regression models for completing the surveys are presented in Table 2, and are graphically illustrated in Additional file 1: Fig. S1. Regression model 1 (survey incentive, survey length, follow-up waves and field study incentive) indicated that a survey was more likely to be completed if including a $2 cash incentive compared to a gift card of any value (OR = 2.792), and if 3 follow-up waves were issued compared to no follow-ups (OR = 2.121). Survey length and field study incentive had no significant effect on survey completion rate. The inclusion of noise exposure category as a predictor (model 2) revealed results similar to that of model 1, with higher response rates for the $2 cash incentive (OR = 2.798) and 3 follow-up waves (OR = 2.120), but there was no effect of noise exposure or direction on survey completion rate.

**Table 2:** Results of the regression models for recipients completing the survey (including only deliverable surveys)

Model and test relative to intercept-only model	Variable	Fixed effects			Variable level	Completing survey		
		df	Wald χ^2^	*p*-value		*p*-value	OR	95% CI
Model 1 χ^2^(6, *n* = 3576) = 158.793, *p* < 0.0001	**Survey incentive**	**1**	**11.599**	**< 0.001**	Gift card	Ref		
					**$2**	**< 0.001**	**2.792**	**1.546–5.041**
	Survey length	2	2.569	0.277	Short	Ref		
					Medium	0.752	0.927	0.579–1.484
					Long	0.139	0.730	0.482–1.107
	**Follow-up waves**	**2**	**9.627**	**0.008**	0	Ref		
					2	0.114	1.530	0.903–2.591
					**3**	**0.005**	**2.121**	**1.250–3.597**
	Field study incentive	1	0.150	0.699	150	Ref		
					200	0.699	0.936	0.671–1.306
Model 2 χ^2^(11, *n* = 3576) = 162.574, *p* < 0.0001	**Survey incentive**	**1**	**11.643**	**< 0.001**	Gift card	Ref		
					**$2**	**< 0.001**	**2.798**	**1.550–5.054**
	Survey length	2	2.505	0.286	Short	Ref		
					Medium	0.759	0.929	0.580–1.488
					Long	0.144	0.733	0.483–1.112
	**Follow-up waves**	**2**	**9.592**	**0.008**	0	Ref		
					2	0.114	1.530	0.903–2.592
					**3**	**0.005**	**2.120**	**1.249–3.596**
	Field study incentive	1	0.170	0.680	150	Ref		
					200	0.680	0.932	0.668–1.301
	Noise exposure category	4	3.397	0.494	< 40	Ref		
					40–45	0.562	0.907	0.651–1.263
					45–50	0.306	0.839	0.599–1.175
					50–55	0.671	1.073	0.776–1.484
					> 55	0.594	1.093	0.787–1.519
	Direction	1	1.073	0.300	West	Ref		
					East	0.538	0.936	0.758–1.156

All analyses excluded surveys that could not be delivered for any reason

*df* Degrees of freedom, *OR* Odds Ratio, *CI* Confidence Interval, *Ref* Reference category

Statistically significant (*p* < 0.05) results are indicated with bold typeface

### Interest

Out of 407 completed surveys, 237 respondents (58.2%) were interested in participating in the field study. Regression models for interest, calculated only using data from completed surveys, are given in Table 3, and are graphically illustrated in Additional file 1: Fig. S1.

**Table 3:** Results of the regression models for respondent interest in participating in the field study

Model and test relative to intercept-only model	Variable	Fixed effects			Variable level	Interest in field study		
		df	Wald χ^2^	p-value		p-value	OR	95% CI
Model 1 χ^2^(6, *n* = 402) = 6.885, *p* = 0.332	Survey incentive	1	2.106	0.147	Gift card	Ref		
					$2	0.147	0.417	0.128–1.359
	Survey length	2	2.628	0.269	Short	Ref		
					Medium	0.819	1.111	0.452–2.733
					Long	0.233	0.621	0.284–1.358
	Follow-up waves	2	1.735	0.420	0	Ref		
					2	0.366	1.595	0.581–4.384
					3	0.811	1.130	0.414–3.090
	Field study incentive	1	0.001	0.971	150	Ref		
					200	0.971	1.011	0.550–1.861
Model 2 χ^2^(11, *n* = 402) = 20.832, *p* = 0.035	Survey incentive	1	2.095	0.148	Gift card	Ref		
					$2	0.148	0.408	0.121–1.373
	Survey length	2	2.854	0.240	Short	Ref		
					Medium	0.753	1.158	0.463–2.899
					Long	0.234	0.615	0.277–1.369
	Follow-up waves	2	1.564	0.457	0	Ref		
					2	0.422	1.529	0.543–4.310
					3	0.876	1.086	0.388–3.038
	Field study incentive	1	0.010	0.921	150	Ref		
					200	0.921	0.969	0.519–1.808
	**Noise exposure category**	**4**	**10.830**	**0.029**	< 40	Ref		
					40–45	0.311	0.721	0.383–1.358
					45–50	0.150	1.619	0.841–3.118
					*50–55*	*0.072*	*1.775*	*0.949–3.318*
					> 55	0.171	1.558	0.826–2.940
	Direction	1	2.049	0.152	West			
					East	0.152	0.738	0.487–1.119
Model 3 χ^2^(17, *n* = 359) = 63.308, *p* < 0.0001	*Survey incentive*	*1*	*3.719*	*0.054*	Gift card	Ref		
					*$2*	*0.054*	*0.245*	*0.059–1.023*
	Survey length	2	1.659	0.436	Short	Ref		
					Medium	0.873	1.086	0.396–2.973
					Long	0.330	0.647	0.270–1.553
	Follow-up waves	2	1.461	0.482	0	Ref		
					2	0.228	2.153	0.619–7.489
					3	0.332	1.851	0.534–6.421
	Field study incentive	1	0.164	0.685	150	Ref		
					200	0.685	1.160	0.565–2.381
	*Noise exposure category*	*4*	*8.904*	*0.064*	< 40	Ref		
					40–45	0.803	0.909	0.430–1.924
					45–50	0.114	1.846	0.863–3.949
					**50–55**	**0.029**	**2.304**	**1.088–4.875**
					> 55	0.132	1.768	0.842–3.713
	Direction	1	0.642	0.423	West	Ref		
					East	0. 423	0.823	0.511–1.326
	Sex	1	0.961	0.327	Female	Ref		
					Male	0. 327	0.774	0.464–1.202
	**Age category**	**5**	**33.150**	**< 0.0001**	< 30	Ref		
					*30–39*	*0.073*	*0.140*	*0.016–1.202*
					**40–49**	**0.029**	**0.094**	**0.011–0.781**
					**50–59**	**0.010**	**0.065**	**0.008–0.525**
					**60–69**	**0.001**	**0.032**	**0.004–0.257**
					**≥70**	**< 0.001**	**0.022**	**0.003–0.183**

All analyses excluded surveys that could not be delivered for any reason

*df* Degrees of Freedom, *OR* Odds Ratio, *CI* Confidence Interval, *Ref* Reference category

Statistically significant (*p* < 0.05) results are indicated with bold typeface. Results of borderline statistical significance (*p* = 0.05–0.1) are indicated with italic typeface

The crude model (model 1) was not significantly different from the intercept-only model.

In the fully adjusted regression model 3, residents exposed to 50–55 dB *L*_night_ were more interested in taking part than those exposed to < 40 dB (OR = 2.304). There was a significant effect of age, with a monotonic decrease in the odds of interest in the field study with increasing age. There was also a statistically borderline effect (*p* = 0.054) of survey incentive, whereby recipients of the $2 cash incentive were less likely to be interested in the field study (OR = 0.245).

No effects of survey incentive, survey length, number of follow-up waves or the field study participation incentive were found.

### Participation

Among respondents interested in the field study, 79 respondents (19.4% of all completed surveys, 33.3% of those interested) met the eligibility criteria. Of those interested and eligible, 37 respondents (9.1% of completed surveys, 15.6% of those interested) were enrolled into the field study. Regression models for participating in the field study, calculated only using data from completed surveys, are given in Additional file 1: Table S1 and illustrated in Additional file 1: Fig. S1. In no models were any statistically significant effects of survey incentive, survey length, follow-up waves, field study incentive, age or sex found for the likelihood that respondents would participate in the field study.

### Questionnaire completion and field study participation probabilities

Probabilities of completing the survey and participating in the field study were calculated using regression model 1. The probability of surveys being completed for each observed combination of survey incentive, survey length and follow-up waves are given in Table 4. The more follow-up waves were sent and the shorter the survey length, the more likely it was to receive a completed survey, with a response rate of 21.7% for survey rounds with 3 follow-up waves, a short survey and a $2 cash incentive.

**Table 4:** Predicted probability and 95% confidence intervals (CI) of receiving a completed survey

Sample size (n)	Probability of completing survey and 95% CIs (%)	Follow-up waves	Survey length	Survey incentive
207	21.7 (16.6–27.9)	3	Short	$2
210	20.5 (15.6–26.5)	3	Medium	$2
1041	16.3 (14.2–18.7)	3	Long	$2
805	12.0 (10.0–14.5)	2	Long	$2
219	8.2 (5.2–12.7)	0	Long	$2
1094	3.1 (2.2–4.3)	0	Long	Gift card
Total = 3576				

Data stratified by number of follow-up waves, survey length and survey incentive. Data calculated excluding non-deliverable surveys

Since the $2 cash incentive was superior to gift cards for receiving completed surveys, and therefore likely a more representative sample, we restricted analysis of field study participation to rounds where only the cash incentive was used (rounds 6–17). The probability of respondents participating in the field study for each combination of survey length, follow-up waves and field study incentive, are given in Table 5. We calculated probabilities based on both the total number of surveys mailed and from among completed surveys only. Since the field study incentive of $100 was offered only in rounds 1–5, probabilities are presented for incentive amounts of $150 and $200 only. The shorter the survey length, the more likely it was for a respondent to participate in the field study. Generally, participation was more likely with more follow-up waves and with the lower field study incentive, although there may be some confounding among these variables due to the unbalanced design.

**Table 5:** Predicted probability and 95% confidence intervals (CI) of a recipient participating in the field study

Follow-up waves	Survey length	Field study participation amount	Sample size (n)	Probability of participating in field study (% with 95% CIs)^a^	Probability of participating among survey respondents (% with 95% CIs)^b^
3	Short	$150	207	2.9 (1.3–6.3)	13.3 (6.1–26.7)
3	Medium	$150	210	2.4 (1.0–5.6)	11.6 (4.9–25.1)
3	Long	$150	427	2.1 (1.1–4.0)	12.5 (6.6–22.3)
2	Long	$200	805	1.0 (0.5–2.0)	8.2 (4.2–15.6)
3	Long	$200	614	0.8 (0.3–1.9)	5.1 (2.1–11.7)
0	Long	$200	219	0.5 (0.1–3.2)	5.6 (0.8–30.7)
			Total = 2482		

Data stratified by number of follow-up waves, survey length, and field study participation amount. Data calculated excluding non-deliverable surveys and gift card incentive rounds. Data ordered from highest to lowest probability of participating in field study

^a^Based on total number of surveys mailed (*n* = 2482)

^b^Based only on completed surveys (*n* = 407)

### Cost effectiveness

In rounds 1–5, the gift card amount was randomized among respondents, so we used the mean cost of the possible $2, $5 and $10 amounts ($5.67) in the cost calculations. In rounds 6–17, 12.4% of initial survey waves were non-deliverable and returned to us with the $2 cash incentive still included. For each individual survey that was completed, an average of $0.248 was recouped from these non-deliverable initial waves, and accounted for in the cost calculations. The costs for each individual survey and follow-up wave mailed out, the total cost per individual and the resulting total cost to receive a single completed survey are presented in Table 6, stratified by the different survey sampling protocols and using the calculation procedure specified in the Additional file 1. The number of surveys sent out to receive a single response are the reciprocals of the response probabilities in Table 4. These data do not account for any associated personnel costs.

**Table 6:** Survey sampling cost effectiveness

Sampling protocol			Surveys sent to receive 1 response (n)^a^	Surveys sent to recruit 1 participant (n)^a,d^	Costs ($)							
Follow-up waves (n)	Survey length	Survey incentive			Initial wave	Follow-up wave 1	Follow-up wave 2	Follow-up wave 3	Total per mailed individual	Per response received^a^	Total to receive 1 response^b^	Recruit 1 participant^b,d^
3	Short	$2	4.61	50.7	3.01	0.70	1.01	1.01	5.74	26.44	28.89	317.51
3	Medium	$2	4.88	53.6	3.09	0.70	1.09	1.09	5.96	29.09	31.84	349.88
0	Long	$2	12.20	134.1	3.09	–	–	–	3.09	37.65	39.54	434.48
3	Long	$2	6.13	67.4	3.09	0.70	1.09	1.09	5.96	36.59	39.99	439.50
2	Long	$2	8.33	91.5	3.09	0.70	1.09	–	4.88	40.64	44.01	483.66
0	Long	Gift card	32.26	354.5	1.09	–	–	–	1.09	40.83^c^	46.81^c^	503.38

Data ordered from most to least cost effective method to receive a single completed survey

^a^Assumes 100% delivery rate

^b^Assumes 87.6% delivery rate and, if applicable, $0.248 recouped from non-deliverable initial survey waves

^c^Includes a mean gift card cost of $5.67

^d^Assumes 9.1% participation rate from completed surveys across all survey mailing rounds, independent of mailing protocol. Does not include cost for actual participation in the field study ($150 or $200)

## Discussion

We evaluated the effectiveness of different survey completion incentives, survey length and number of follow-up waves on survey response rates. A $2 cash incentive almost tripled the odds of receiving a completed survey compared to a gift card. Sending three follow-up waves after the initial mailing more than doubled the odds compared to sending no follow-up. There was no significant effect of any of the assessed variables on the odds of respondents participating in the field study.

### Delivery and response rates

The delivery rate was lower for surveys sent to named individuals, perhaps due to the mail carrier not delivering if the name on the envelope did not match a name at the address despite the appended “or Current Resident”, but this was more than offset by higher response rates among those named addressees. This increased response rate when personalizing the surveys is generally in agreement with previous research. A meta-analysis of 14 trials including over 12,000 participants found that the inclusion of names on health survey letters increased the odds of response by one fifth [29]. A later study however found that addressing surveys to named individuals significantly increased the response rate to reminder letters, but the increased response rate to the initial survey waves was not significant, although in this study of 1000 survey recipients the absence of significance could be due to insufficient power [30]. As well as personalization, the higher response rate could be in part due to the removal of “School of Medicine” and “Department of Psychiatry” from the envelope, since psychiatry as a medical profession continues to suffer from public stigma [31]. We would not anticipate the change in envelope size to influence response [32].

A total response rate of 11.4% is lower than rates of 30–76% for postal surveys on aircraft noise annoyance in Europe and East Asia that were reported in a recent systematic review [33]. Our response rate is however in line with some more general attitudinal surveys [30, 34]. Possible reasons for non-response in our sample might include concerns about privacy and confidentiality despite assurances given in the introduction letter [35], illiteracy or language issues [36] or lack of interest in the survey topic or low community engagement [37]. In the United States, 37.6 million people speak Spanish at home [38], and including Spanish language surveys along with the English versions could improve response rates among this population without lowering response rates from non-Spanish speakers [39].

We received the majority of responses by mail, at a ratio of around 3:1 compared to online response. There is inconsistency among earlier studies regarding the influence of response mode, with some reporting higher response rates for paper surveys compared to online surveys e.g. [34, 40], and others finding an increased preference for completing questionnaires electronically e.g. [41]. We do not know whether those who completed our survey online would have returned it by post if the online option was not available, or vice versa for respondents who completed the survey by mail, and therefore cannot draw any conclusions regarding the optimal choice if only one survey mode were to be used in future studies. Providing multiple response modes is however preferable, as this an effective method to improve overall survey response and representativeness when implemented correctly [1].

We have used survey completion rates as the primary indicator of success of the different mailing strategies, but lack a true measure of nonresponse error, which precludes firm conclusions regarding the effectiveness of the different mailing strategies for improving representativeness of the sample population. Offering web and mail response modes concurrently, rather than sequentially, may have reduced the overall response rate [1], although evidence is mixed [42]. Hypothesized reasons for this effect include, firstly, increased complexity in the decision to respond by introducing the choice of response mode; secondly, respondents choosing to respond online but never actually doing so since it involves a break in the response process; and thirdly sample members attempting to respond by web but not completing the survey due to computer or internet connectivity issues [43]. Initial mail contact offering a web-based response, and withholding paper surveys until later mailing rounds, may increase response rates compared to a paper-only method, but without significantly improving respondent representativeness [44]. A higher response rate, while not necessarily indicating greater respondent representativeness or data quality [7–9], may at least reduce the risk of nonresponse bias [1]. The pilot study presented in the current paper is a preceding step towards a national study of the potential effects of aircraft noise on sleep, and this future study offers the opportunity to more rigorously address nonresponse bias. One approach that has been widely used is comparing respondent characteristics to known characteristics of the whole population of interest [6, 45], in this case residents exposed to a certain minimum level of aircraft noise, using demographic data at the census tract level from the decennial U. S. Census [46] and the American Community Survey [25].

### Effect of different sampling protocols

Our findings on the effectiveness of different surveying strategies are in good agreement with the existing literature. For instance, a previous meta-analysis found that response to health research postal questionnaires could be improved by implementing repeat mailing strategies and, to a lesser degree, using shorter questionnaires [14]. In particular, the effectiveness of follow-ups on increasing response is rather well established in the existing literature [13, 47]. Similarly, we attained the highest response rate when using the most intensive follow-up strategy, but observed no significant increases in response when shortening the questionnaire length.

According to the “continuum of resistance” model, the greater the number of contacts that are required before receiving a response, the more similar that eventual respondent is to a non-respondent [48]. Our observed increase in response with an increasing number of follow-up contacts in the current data could therefore indicate increasing representativeness of the sampled population. The same is not necessarily true for our higher completion rates when using monetary incentives however. The use of incentives, particularly monetary incentives, increases response rates to all survey modes [49], but if they are equally effective across all sample members then they are unlikely to affect nonresponse bias [50].

Only the mailing rounds with gift card incentives offered $100 for field study participation, and only the rounds with cash incentives offered $150 or $200 for field study participation, which is a limitation of the study design. The almost three times higher odds in survey response when we used a cash incentive is most plausibly due to the $2 cash outperforming the gift card as an incentive, rather than the difference in field study participation incentives. This is supported by the lack of observed differences in response rates between $150 and $200 field study incentives, the fact that monetary incentives have previously been found to outperform non-monetary incentives and that prepaid incentives outperform promised incentives [13, 51–54]. Furthermore, completion of the survey did not obligate field study participation, so we did not anticipate that field study compensation would influence survey response rates.

Older people are, for multiple reasons, frequently more difficult to recruit into experimental studies [55]. Accordingly, younger people in our survey sample were more interested in taking part in the field study. When endeavoring to recruit evenly distributed age groups in studies, oversampling from the target population might be needed.

The lack of significant difference in the odds of participation for different field study compensation amounts could suggest that the participants had more self-determined motivational traits [56], and/or that general interest in the research was a primary reason for taking part rather than financial interests alone. The hypothesis for personal interest is supported by the doubled odds of interest in the study for respondents exposed to 50–55 dB noise relative to the lowest noise category. Populations exposed to higher noise levels could be expected, through personal experience, to be more acutely aware of the issue of nocturnal aircraft noise, and therefore more willing to contribute to research on its effects. The odds in the highest exposure category (> 55 dB) were not significantly higher than in the lowest category, which on one hand would not substantiate the idea for greater interest among those most affected, but could alternatively be explained by the most adversely affected people self-selecting themselves out of the area by moving to a quieter neighborhood.

Although rounds 1–5 offered $100 for field study participation, these mailing rounds also exclusively included gift cards as survey incentives, and so we cannot draw conclusions regarding differences in participation rates between $100 and $150/$200 amounts. Furthermore, the absence of significant findings could result from insufficient statistical power, since only 37 subjects eventually participated in the field study.

The highest probability of field study participation, achieved with the short survey - although not statistically significant - may reflect a modest advantage of using a reduced survey length. On the other hand, the short survey required additional telephone contact, which may be the cause of a potential higher participation likelihood, rather than the short survey per se.

### Cost effectiveness

The most inexpensive sampling protocol had the lowest response rate, with the consequence that it was the least effective approach in terms of the financial cost to receive one completed survey. Conversely, the three sampling protocols with three follow-up waves were the most expensive, but when using the short and medium length survey were the most cost effective approaches owing to their increased response rates. The short survey was the most cost effective in terms of materials due to a slightly lower cost and a higher response rate. We required additional telephone contact with the short survey respondents to obtain further information regarding field study eligibility, but since personnel costs were not included, this approach may not truly be the most cost effective approach overall for field study recruitment.

Three follow-up waves approximately doubled the response rate compared to sending no follow-up. The additional cost of those follow-up waves ($2.88 for long surveys) was comparable with the cost of mailing a new long survey to a new household with no follow-ups ($3.09), hence both approaches could be anticipated to yield similar response rates at similar costs. This is consistent with findings reported by Mayfield et al. [57]. Furthermore, late responders who did not respond to initial contact may be more similar to non-respondents [58], so increasing the response rate from initial non-responders can help to minimize bias and increase the representativeness of the sample.

### Limitations and future research

A weakness of this study is the somewhat limited number of respondents. Although we sampled 4080 households, many of these mailings used strategies that were especially ineffectual at eliciting response. For instance, when using gift cards and no follow-up contact, reflecting almost a third of all deliverable surveys, the response rate was only 3.1%. On the one hand, the size of the effects between the most and least effective mailing strategies despite the modest sample sizes and width of the confidence intervals helps to demonstrate the inferiority of the promise of gift cards with no follow-up contact compared to alternative approaches. On the other hand, data in the models for interest and participation in the field study stem from only 407 respondents, meaning the results should be interpreted with caution. However, it is noteworthy that this number of respondents is comparable to or exceeds sample sizes from some recent survey studies on the effects of aircraft noise on sleep [59].

The survey rounds were not issued concurrently, but the earlier rounds were sent in autumn, the middle rounds were sent in winter or spring and the final rounds were sent in early summer. We cannot totally exclude there are subsequent effects on response rate, perhaps because residents were not home at certain times of year, or that there are seasonal effects influencing the predisposition of an individual to complete the questionnaire [60].

The study design was not perfectly balanced, so we cannot conclude whether increasing the field study compensation from $100 to $150 or $200 would have affected recruitment. To avoid possible confounding, an alternative study design, but with additional expense, could involve a 2 × 2 × 3 × 3 factorial design with the factors of pre−/post-completion incentive, $2/gift card incentive, short/medium/long survey length and 0/2/3 follow-up waves.

## Conclusions

Prepaid cash incentives and sending follow-up reminder and survey waves were an effective method of improving response rates to postal questionnaires. Although no factors of the different sampling protocols improved the probability of a respondent participating in the field study per se, using a pre-issued cash incentive and sending more follow-up waves, and subsequently improving response rates and achieving higher numbers of people from which to recruit, may be an effective strategy for improving recruitment into field studies.

## Supplementary information

**Additional file 1.** A. Questionnaire instruments. B. Cost estimate calculations. C. Field study participation. D. Figures.

## Data Availability

The datasets generated and analyzed during the current study are not publicly available due to containing information that could potentially identify study participants, but de-identified data are available from the corresponding author on reasonable request.

## Abbreviations

ATL: Hartsfield-Jackson Atlanta International Airport
CI: Confidence interval
dB: Decibel
FAA: Federal Aviation Administration
*L*_night_: Outdoor sound pressure level from aircraft during the night (23:00–07:00)
OR: Odds ratio
