# Supplementary material for: Effectiveness of incentives and follow-up on increasing survey response rates and participation in field studies
Source: BMC Med Res Methodol. 2019 Dec 5;19:230. doi: 10.1186/s12874-019-0868-8 (PMC6896692; doi:10.1186/s12874-019-0868-8)
Supplement: Supplementary file 1 — Additional file 1. A. Questionnaire instruments. B. Cost estimate calculations. C. Field study participation. D. Figures. [file 12874_2019_868_MOESM1_ESM.pdf]

Effectiveness of incentives and follow-up on increasing survey response rates and participation in field studies

Supplementary Online Materials

Authors and affiliations Michael G. Smith[1*], Maryam Witte[1], Sarah Rocha[1] and Mathias Basner[1]

[1] Unit for Experimental Psychiatry, Division of Sleep and Chronobiology, University of Pennsylvania Perelman School of Medicine, Philadelphia, PA, USA

*Corresponding author: Michael G. Smith
Michael.smith@pennmedicine.upenn.edu
Unit for Experimental Psychiatry, Division of Sleep and Chronobiology, University of Pennsylvania Perelman School of Medicine, 1020 Blockley Hall, 423 Guardian Drive

This supplement provides ancillary methods and results to the main text:

A. Questionnaire instruments

Complete versions of the short, medium and long questionnaires are given on p. 2, pp. 3-4 and pp. 5-8 respectively.

B. Cost estimate calculations

Formulae used to estimate the costs to receive one completed survey and to recruit one participant into the field study are given on p. 9.

C. Field study participation

Results of the regression models for the odds of a mailed individual eventually participating in the field study are given in Table S1 (p. 10). Data are visualized for each predictor variable in Figure S1 (p. 11).

D. Figures

Figure S1 (p. 11) presents the odds ratios of completing the survey, interest in the field study and participating in the field study. Results are given for each independent predictor variable in the fully adjusted regression models: Model 2 for survey completion and Model 3 for interest and participation in the field study. The presented results are from Table 2 and Table 3 in the main article, and Table S1 in this Supplemental Material (p. 10).

Short questionnaire

Q1. During the **past month**, how would you rate your sleep quality overall?

Very Good	Fairly Good	Fairly Bad	Very Bad
☐	☐	☐	☐

Q2. In general, would you say your health is…?

Poor	Fair	Good	Very Good	Excellent
☐	☐	☐	☐	☐

Q3. Rate how strongly you agree or disagree to the statement: I am sensitive to noise

Strongly Disagree 1	2	3	4	5	Strongly Agree 6
☐	☐	☐	☐	☐	☐

Q4. Thinking about the **last 12 months** or so, when you were here at home, how much was your sleep disturbed by noise from the following sources?

	Not at all	Slightly	Moderately	Very	Extremely
**Q4a**. Road Traffic	☐	☐	☐	☐	☐
**Q4b**. Trains	☐	☐	☐	☐	☐
**Q4c**. Aircraft	☐	☐	☐	☐	☐
**Q4d**. Neighbors	☐	☐	☐	☐	☐

Q5. Are you Hispanic or Latino? ☐ Yes ☐ No ☐ Prefer Not to Answer

Q6. What race do you consider yourself to be? (mark all that apply)

- ☐ American Indian or Alaska Native
- ☐ Asian
- ☐ Black or African American
- ☐ Prefer Not to Answer
- ☐ Native Hawaiian or Other Pacific Islander
- ☐ White
- ☐ Other (please specify):________________________

Q7. Gender: ☐ Male ☐ Female

Q8. Age: ________________________(years)

Medium questionnaire

Q1. During the **past month**, how would you rate your sleep quality overall?

Very Good	Fairly Good	Fairly Bad	Very Bad
☐	☐	☐	☐

Q2. How often have you have taken medicine (prescribed or "over the counter") to help you sleep in the **past month**?

Not during the past month	Less than once a week	Once or twice a week	Three or more times a week
☐	☐	☐	☐

Q3. How often in the **past month** have you done the following because of noise when trying to sleep at home?

	Never 1	Rarely 2	Sometimes 3	Often 4	Always 5
**Q3a**. Wear earplugs or headphones	☐	☐	☐	☐	☐
**Q3b**. Turn on the TV	☐	☐	☐	☐	☐
**Q3c**. Turn on music	☐	☐	☐	☐	☐
**Q3d**. Close windows	☐	☐	☐	☐	☐
**Q3e**. Use a sound machine	☐	☐	☐	☐	☐
**Q3f**. Turn on a fan	☐	☐	☐	☐	☐

Q4. Rate how strongly you agree or disagree to the statement: I am sensitive to noise

Strongly Disagree 1	2	3	4	5	Strongly Agree 6
☐	☐	☐	☐	☐	☐

Q5. Thinking about the **last 12 months** or so, when you were here at home, how much was your sleep disturbed by noise from the following sources?

	Not at all	Slightly	Moderately	Very	Extremely
**Q5a**. Road Traffic	☐	☐	☐	☐	☐
**Q5b**. Trains	☐	☐	☐	☐	☐
**Q5c**. Aircraft	☐	☐	☐	☐	☐
**Q5d**. Neighbors	☐	☐	☐	☐	☐

Q6. In general, would you say your health is…?

Poor	Fair	Good	Very Good	Excellent
☐	☐	☐	☐	☐

Q7. Have you ever been diagnosed by a health professional with any of the following sleep disorders?

☐ Sleep Apnea ☐ Narcolepsy ☐ Restless Leg Syndrome

☐ Periodic Limb Movement Syndrome ☐ Insomnia ☐ None

☐ Other (please specify): ______________________________

Q8. Do you have any problems or difficulties with your sense of hearing? ☐ Yes ☐ No

Q9. Have you ever been diagnosed by a health professional with the following conditions (mark all that apply)?

☐ Hypertension/ High blood pressure ☐ Arrhythmia/ Irregular heartbeat ☐ None

Q10. If currently employed, does your job require overnight shift work?

(Overnight shift work refers to work for at least 4 hours between 12 a.m. midnight to 6 a.m. in the morning) ☐ Yes ☐ No

Q11. How long have you lived at your current residence

**Less than 1 year**	**1-5 years**	**5-10 years**	**More than 10 years**
▼	▼	▼	▼
☐	☐	☐	☐

Q12. Are there children in this household under the age of 5? ☐ Yes ☐ No

Q13. Are you Hispanic or Latino? ☐ Yes ☐ No ☐ Prefer Not to Answer

Q14 What race do you consider yourself to be? (mark all that apply)

☐ American Indian or Alaska Native ☐ Native Hawaiian or Other Pacific Islander

☐ Asian ☐ White

☐ Black or African American ☐ Other (please specify):______________________

☐ Prefer Not to Answer

Q15. Gender: ☐ Male ☐ Female **Q16.** Age: ____________________(years)

Q17. What is your height? __________feet__________inches

Q18. What is your weight? __________________________lbs

Long questionnaire

**Q1a**. During the **past month**, at what time have you usually gone to bed on weekdays or workdays?	____________________(HH:MM AM/PM)
**Q1b**. During the **past month**, at what time have you usually woken up on weekdays or workdays?	____________________(HH:MM AM/PM)
**Q1c**. During the **past month**, how much sleep did you usually get on weekdays or workdays?	____________________(Hours)

Q2. During the **past month**, how would you rate your sleep quality overall?

Very Good	Fairly Good	Fairly Bad	Very Bad
▼ ☐	▼ ☐	▼ ☐	▼ ☐

Q3. For the following questions, select the response that best reflects how often the following occurred during the **past month.**

	Not during the past month ▼	Less than once a week ▼	Once or twice a week ▼	Three or more times a week ▼
**Q3a**. You had trouble sleeping because you cannot get to sleep within **30 minutes**?	☐	☐	☐	☐
**Q3b**. You had trouble sleeping because you wake up in the middle of the night or early morning?	☐	☐	☐	☐
**Q3c**. You have taken medicine (prescribed or "over the counter") to help you sleep?	☐	☐	☐	☐
**Q3d**. You had trouble staying awake while driving, eating meals, or engaging in social activity?	☐	☐	☐	☐

Q4. How often in the **past month** have you done the following because of noise when trying to sleep at home?

	Never 1 ▼	Rarely 2 ▼	Sometimes 3 ▼	Often 4 ▼	Always 5 ▼
**Q4a**. Wear earplugs or headphones	☐	☐	☐	☐	☐
**Q4b**. Use alcohol	☐	☐	☐	☐	☐
**Q4c**. Use medication	☐	☐	☐	☐	☐
**Q4d**. Turn on the TV	☐	☐	☐	☐	☐
**Q4e**. Turn on music	☐	☐	☐	☐	☐
**Q4f**. Close windows	☐	☐	☐	☐	☐
**Q4g**. Use a sound machine	☐	☐	☐	☐	☐
**Q4h**. Turn on a fan	☐	☐	☐	☐	☐

Q5. For the following statements respond how strongly you agree or disagree.	Strongly Disagree 1	2	3	4	5	Strongly Agree 6
Q5a. I am easily awakened by noise	☐	☐	☐	☐	☐	☐
Q5b. I get used to most noises without much difficulty	☐	☐	☐	☐	☐	☐
Q5c. I find it hard to relax in a place that is noisy	☐	☐	☐	☐	☐	☐
Q5d. I am good at concentrating no matter what is going on around me	☐	☐	☐	☐	☐	☐
Q5e. I get mad at people who make noise that keeps me from falling asleep or getting work done	☐	☐	☐	☐	☐	☐
Q5f. I am sensitive to noise	☐	☐	☐	☐	☐	☐

Q6. Thinking about the **last 12 months** or so, when you were here at home, how much was your sleep disturbed by noise from the following sources?

	Not at all	Slightly	Moderately	Very	Extremely
Q6a. Road Traffic	☐	☐	☐	☐	☐
Q6b. Trains	☐	☐	☐	☐	☐
Q6c. Aircraft	☐	☐	☐	☐	☐
Q6d. Industries/Factories	☐	☐	☐	☐	☐
Q6e. Construction	☐	☐	☐	☐	☐
Q6f. Neighbors	☐	☐	☐	☐	☐
Q6g. Air Conditioner	☐	☐	☐	☐	☐

Q7. Thinking about the **last 12 months** or so, when you are here at home, how much does noise from each of the following bother, disturb, or annoy you?

	Not at all	Slightly	Moderately	Very	Extremely
Q7a. Road Traffic	☐	☐	☐	☐	☐
Q7b. Trains	☐	☐	☐	☐	☐
Q7c. Aircraft	☐	☐	☐	☐	☐
Q7d. Industries/Factories	☐	☐	☐	☐	☐
Q7e. Construction	☐	☐	☐	☐	☐
Q7f. Neighbors	☐	☐	☐	☐	☐
Q7g. Air Conditioner	☐	☐	☐	☐	☐

Q8. In general, would you say your health is…?

Poor	Fair	Good	Very Good	Excellent
☐	☐	☐	☐	☐

Q9. Have you ever been diagnosed by a health professional with any of the following sleep disorders?

- ☐ Sleep Apnea
- ☐ Narcolepsy
- ☐ Restless Leg Syndrome
- ☐ Periodic Limb Movement Syndrome
- ☐ Insomnia
- ☐ None
- ☐ Other (please specify): ______________________________

Q10. Do you have any problems or difficulties with your sense of hearing? ☐ Yes ☐ No

**Q11a**. Have you ever been diagnosed by a health professional with the following conditions (mark all that apply)?	**Q11b**. If you have been diagnosed with a condition listed under Q11a, have you been treated for the condition in the **past month?**
☐ Hypertension/High blood pressure	☐ Yes ☐ No
☐ Chronic headaches/Migraines	☐ Yes ☐ No
☐ Arrhythmia/Irregular heartbeat	☐ Yes ☐ No
☐ Heart disease	☐ Yes ☐ No
☐ Stomach ulcer	☐ Yes ☐ No
☐ Diabetes	☐ Yes ☐ No
☐ None of the above	

Q12. What is your marital status?

- ☐ Single
- ☐ Married
- ☐ Widowed
- ☐ Separated
- ☐ Divorced
- ☐ Domestic Partners

Q13. What was your total household income last year?

- ☐ < $25,000
- ☐ $25,000-$50,000
- ☐ $50,000-$75,000
- ☐ $75,000-$100,000
- ☐ $100,000-$150,000
- ☐ >$150,000
- ☐ Prefer Not to Answer

Q14. What is the highest level of education you have completed?

- ☐ Less than High School
- ☐ High School Graduate
- ☐ College Graduate or Higher

Q15. What is your current employment status?

- ☐ Working
- ☐ Unemployed
- ☐ Student
- ☐ Retired
- ☐ Homemaker

Q16. If currently employed, does your job require overnight shift work?

(Overnight shift work refers to work for at least 4 hours between 12 am midnight to 6 am in the morning) ☐ Yes ☐ No

Q17. Are you Hispanic or Latino? ☐ Yes ☐ No ☐ Prefer Not to Answer

Q18. What race do you consider yourself to be? (mark all that apply)

- ☐ American Indian or Alaska Native
- ☐ Native Hawaiian or Other Pacific Islander
- ☐ Asian
- ☐ White
- ☐ Black or African American
- ☐ Other (please specify):________________________
- ☐ Prefer Not to Answer

Q19. Has your current residence received any sound proofing treatment to reduce noise? ☐ Yes ☐ No

Q20. How long have you lived at your current residence?

- ☐ Less than 1 year
- ☐ 1-5 years
- ☐ 5-10 years
- ☐ More than 10 years

Q21. Do you have an air conditioner in your bedroom?

- ☐ No Unit
- ☐ Central Air Conditioner
- ☐ Window Unit

**Q22.** How many people (including yourself) reside in this household?	______________________
**Q23.** How many children in this household are under the age of 5?	______________________

**Q24.** Gender:	☐ Male ☐ Female	**Q25.** Age:	______________________(years)

**Q26.** What is your height?	__________feet__________inches
**Q27.** What is your weight?	__________________________lbs

Cost estimate calculations

$$\#nondeliverable\ surveys = \frac{(nondelivery\ rate)}{delivery\ rate} \times \#surveys\ delivered$$

where *#surveys delivered* is the number of surveys that must be delivered in order to receive one response to the postal survey (Table 6 in main manuscript).

$$reclaimed\ cash\ incentives = \#nondelilverable\ surveys \times \$2$$

$$\begin{aligned} &cost\ estimate, 1\ response \\ &\quad = (\#surveys\ delivered\ \times cost\ of\ single\ mailing) \\ &\quad + (\#nondeliverable\ surveys\ \times cost\ of\ single\ mailing) \\ &\quad - reclaimed\ cash\ incentives \end{aligned}$$

$$\begin{aligned} &cost\ estimate, 1\ participant \\ &\quad = cost\ estimate, 1\ response\ \times {}^{1}\!/_{field\ study\ participation\ rate} \end{aligned}$$

The following example is for short surveys using a \$2 cash incentive and 3 follow-up waves:

Delivery rate = 87.6%; #surveys delivered = 4.61

$$\#nondeliverable\ surveys = \frac{(1 - 0.876)}{0.876} \times 4.61 = 0.65$$

$$reclaimed\ cash\ incentives = 0.65 \times \$2 = 1.3$$

A complete mailing round costs \$5.74:

$$cost\ estimate, 1\ response = (4.61\ \times 5.74) + (0.65\ \times 5.74) - 1.3 = \$\mathbf{28.89}$$

Field study participation rate = 9.1%;

$$cost\ estimate, 1\ participant = 28.89\ \times {}^{1}\!/_{0.091} = \$\mathbf{317.47}$$

The \$0.04 difference between this cost estimate for one participant and the cost estimate in Table 6 is due to rounding of the values in the above calculation. The cost estimates in Table 6 are calculated using non-rounded data.

Table S1 Results of the regression models for recipients participating in the field study (including only completed surveys). All analyses excluded surveys that could not be delivered for any reason. df=Degrees of Freedom. OR=Odds Ratio. CI=Confidence Interval. Ref=Reference category.

Model and test relative to intercept-only model	Variable	Fixed effects			Variable level	Field study participation		
		df	Wald χ^2	p		p-value	OR	95% CI
Model 1 χ^2(6, n=407)=4.707, p=0.582	Survey incentive	1	0.174	0.677	Gift card	Ref		
					$2	0.677	0.608	0.059-6.305
	Survey length	2	0.058	0.971	Short	Ref		
					Medium	0.809	0.855	0.241-3.040
					Long	0.896	0.929	0.307-2.811
	Follow-up waves	2	0.805	0.669	0	Ref		
					2	0.698	1.528	0.179-13.022
					3	0.936	0.914	0.100-8.300
	Field study incentive	1	2.828	0.093	150	Ref		
					200	0.093	0.376	0.120-1.176
Model 2 χ^2(9, n=407)=10.502, p=0.486	Survey incentive	1	0.294	0.588	Gift card	Ref		
					$2	0. 588	0.521	0.049-5.505
	Survey length	2	0.065	0.968	Short	Ref		
					Medium	0.810	0.854	0.236-3.095
					Long	0.843	0.892	0.290-2.748
	Follow-up waves	2	1.012	0.603	0	Ref		
					2	0.628	1.703	0.197-14.691
					3	0.971	0.960	0.104-8.834
	Field study incentive	1	3.254	0.071	150	Ref		
					200	0. 071	0.346	0.109-1.094
	Noise exposure category	4	3.662	0.454	<40	Ref		
					40-45	0.258	0.519	0.166-1.619
					45-50	0.906	1.061	0.399-2.818
					50-55	0.605	0.770	0.285-2.079
					>55	0.142	0.427	0.137-1.330
	Direction	1	1.917	0.166	West	Ref		
					East	0.166	0.607	0.299-1.231
Model 3 χ^2 (17, n=364)=13.496, p=0.702	Survey incentive	1	0.286	0.593	Gift card	Ref		
					$2	0. 593	0.520	0.047-5.730
	Survey length	2	0.011	0.995	Short	Ref		
					Medium	0.919	0.933	0.244-3.569
					Long	0.944	0.959	0.303-3.036
	Follow-up waves	2	1.092	0.579	0	Ref		
					2	0.642	1.687	0.187-15.238
					3	0.935	0.910	0.094-8.817
	Field study incentive	1	3.190	0.074	150	Ref		
					200	0.074	0.344	0.107-1.110
	Noise exposure category	4	3.432	0.488	<40	Ref		
					40-45	0.354	0.570	0.173-1.873
					45-50	0.992	0.995	0.360-2.746
					50-55	0.722	0.828	0.293-2.340
					>55	0.119	0.391	0.120-1.274
	Direction	1	1.877	0.171	West	Ref		
					East	0.171	0.602	0.291-1.245
	Sex	1	0.081	0.776	Female	Ref		
					Male	0.776	0.894	0.411-1.942
	Age category	5	3.223	0.666	<30	Ref		
					30-39	0.906	1.096	0.237-5.064
					40-49	0.696	0.737	0.159-3.410
					50-59	0.624	0.686	0.152-3.093
					60-69	0.722	0.764	0.173-3.368
					≥70	0.173	0.263	0.039-1.793

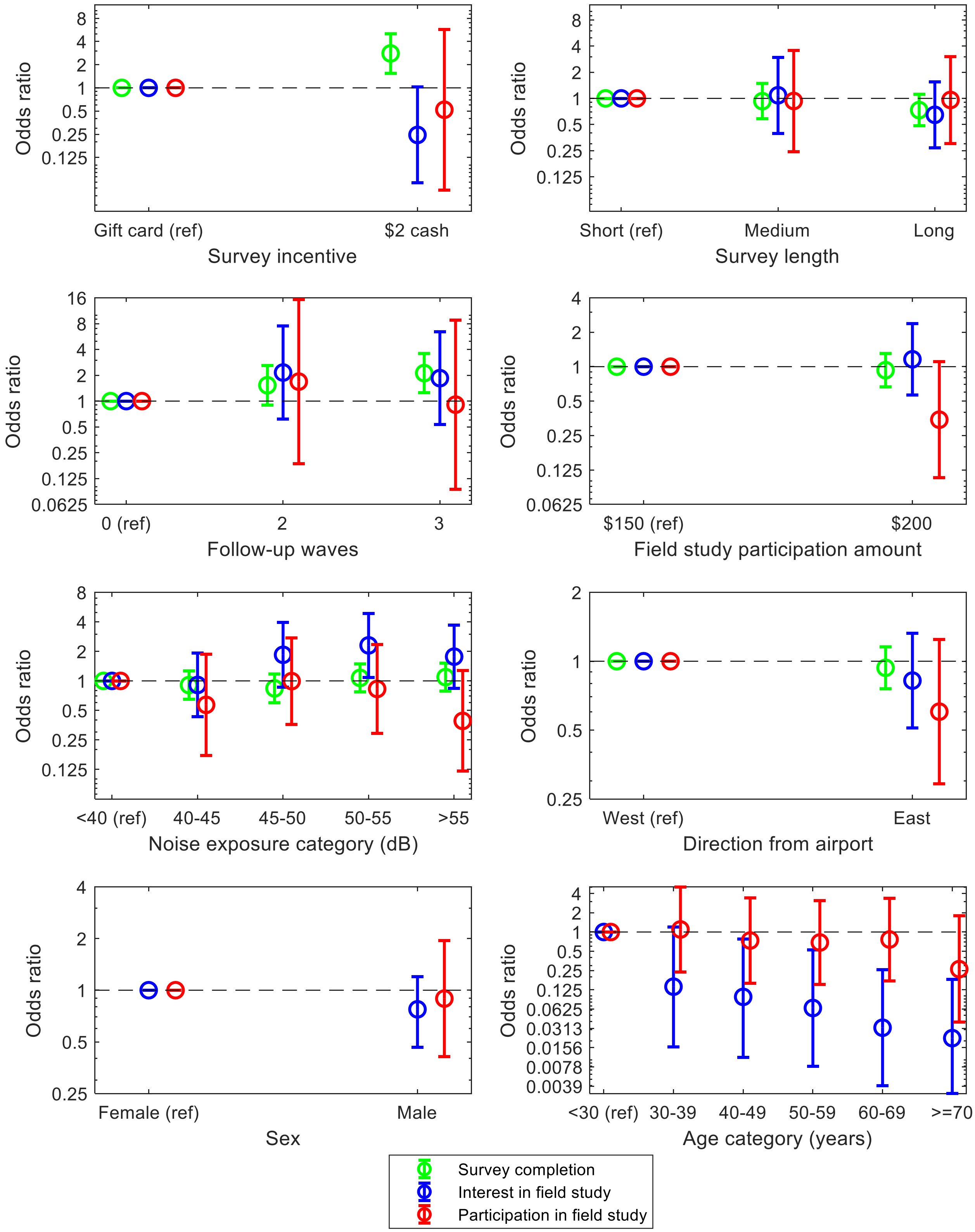

Figure S1 Odds ratios and 95% confidence intervals for the effect of different survey approaches and situational factors on receiving completed surveys (green), eliciting interest in the study (blue) and recruiting a participant into the study (red). The horizontal dashed line indicates the reference value OR=1.0.
